# Understanding patient-reported knowledge of hernia surgery: a quantitative study

**DOI:** 10.1007/s10029-021-02521-6

**Published:** 2021-10-20

**Authors:** D. Rosselló Jiménez, M. López-Cano, V. Rodrigues Gonçalves, M. Verdaguer Tremolosa, J. Saludes Serra, A. Bravo-Salva, J. A. Pereira Rodríguez

**Affiliations:** 1 Geriatric Service, Consorci Sanitari de Terrassa, Terrassa, Barcelona, Spain; 2 Abdominal Wall Surgery Unit, Department of General Surgery, Hospital Universitari Vall d’Hebron, Universitat Autònoma de Barcelona, Passeig Vall d’Hebron 119-129, 08035 Barcelona, Spain; 3 Service of Anesthesia and Resuscitation, Hospital Universitario de Tarragona Joan XXIII, Tarragoa, Spain; 4 Department of General and Digestive Surgery, Hospital Universitario del Mar, Parc de Salut Mar, Barcelona, Spain; 5 Department of Experimental and Health Sciences, Universitat Pompeu Fabra, Barcelona, Spain

**Keywords:** Hernia, Surgery, Abdominal wall, Knowledge, Patient

## Abstract

**Purpose:**

The objective of this study was to gather information on patient-reported knowledge (PRK) in the field of hernia surgery.

**Methods:**

A prospective quantitative study was designed to explore different aspects of PRK and opinions regarding hernia surgery. Patients referred for the first time to a surgical service with a presumed diagnosis of hernia and eventual hernia repair were eligible, and those who gave consent completed a simple self-assessment questionnaire before the clinical visit.

**Results:**

The study population included 449 patients (72.8% men, mean age 61.5). Twenty (4.5%) patients did not have hernia on physical examination. The patient’s perceived health status was “neither bad nor good” or “good” in 56.6% of cases. Also, more patients considered that hernia repair would be an easy procedure (35.1%) rather than a difficult one (9.8%). Although patients were referred by their family physicians, 32 (7.1%) answered negatively to the question of coming to the visit to assess the presence of a hernia. The most important reason of the medical visit was to receive medical advice (77.7%), to be operated on as soon as possible (40.1%) or to be included in the surgical waiting list (35.9%). Also, 46.1% of the patients considered that they should undergo a hernia repair and 56.8% that surgery will be a definitive solution.

**Conclusion:**

PRK of patients referred for the first time to an abdominal wall surgery unit with a presumed diagnosis of hernia was quite limited and there is still a long way towards improving knowledge of hernia surgery.

**Supplementary Information:**

The online version contains supplementary material available at 10.1007/s10029-021-02521-6.

## Introduction

Abdominal wall hernia surgery encompasses repair of very common defects, but has progressively turned into a challenging surgical entity due to complexity of some hernias, wide variety of repair techniques or difficulties in choosing the appropriate prosthetic mesh [1]. On the other hand, evidence-based medicine and shared decision-making (SDM) models have defined a new approach where clinicians and patients make decisions together based on the best scientific evidence available, with consideration of the clinician’s experience and the patient’s preferences and values [2, 3]. However, current definitions of the patient’s values from a patients’ perspective are frequently vague [4], although they may be understood as “the unique preferences, concerns and expectations each patient brings to a clinical encounter and which must be integrated into clinical decision is they are to serve the patient” [5].

Patient-reported outcomes (PRO) are directly reported by the patient without interpretation of the patient’s response by a clinician or anyone else and pertain to the patient’s health, quality of life or functional status associated with healthcare or treatment [6–8]. These outcomes are obtained using self-administered questionnaires before and after surgery, measuring the severity of pain, health-related quality of life, physical limitations, etc., and have emerged as reliable alternatives or complementary information to conventionally reported recurrence rates in hernia research [9]. These health outcomes directly reported by the patient are mainly focused on measuring the patient’s health status or quality of life at a particular moment in time [10]. However, little is known about what kind of knowledge or understanding the patient has of his/her hernia disease at the first medical encounter. As far as we are aware, specific studies on this matter have not been previously published. There is probably a conceptual asymmetry since surgeons and patients do not have to have the same level of information. In addition, preferences, concerns and expectation may be influenced if the patient seeks medical news on the internet before consultation, which may potentially contribute to biased or inaccurate perception of the illness. In this respect, assessment of the patient’s knowledge and understanding about their disease [11], in terms named as patient-reported knowledge (PRK) [12, 13], may be a key factor influencing implementation of the SDM model in clinical practice. Therefore, in general surgery and other subspecialties, such as abdominal wall hernia surgery, incorporation of the PRK in association with other measures such as hernia recurrence rate and PRO can provide structure to more accurately identify patient values [14, 15].

Because of the lack of data of PRK in the setting of surgical repair of abdominal wall defects and considering the exploratory nature of the study, a prospective quantitative study design [16] was selected. The primary objective of the study was to evaluate PRK of patients referred for the first time to an abdominal wall surgery unit for confirmation of a presumed diagnosis of hernia and eventual hernia repair.

## Methods

### Design and participants

Between April 2019 and October 2020, a prospective observational quantitative study was conducted at two large hospitals in Barcelona, Spain. One of the hospitals, Hospital Universitari Vall d’Hebron, is an 1146-bed acute-care teaching hospital serving an urban population of approximately 500,000 people. The second hospital, Hospital del Mar, is a 471-bed acute-care teaching hospital serving an urban population of approximately 300,000 people. All patients older than 18 years of age who were referred for a first visit to the Department of Surgery to one of the two hospitals by specialists in family and community medicine with a presumed diagnosis of hernia were included in the study. Any medical appointment other than a first visit was an exclusion criterion.

Patients were fully informed about the objective and characteristics of the study, and those who voluntarily accepted to take part were requested to sign the written informed consent and to complete a self-assessment study questionnaire. The study protocol was approved by the Clinical Research Ethical Committee of the reference hospital (Hospital Universitari Vall d’Hebron) (code number PR [AG]06/2019).

### Study questionnaire

An ad hoc study questionnaire was designed for the purpose of the study by two of the authors (D.R.J., M.L-C.). Because of the lack of data of PRK in the setting of surgical repair of abdominal wall defects and the exploratory nature of the study survey testing before the study was not done. This was a short and simple self-assessment instrument with a minimum number of items to reduce the patient’s burden and the time needed to complete all questions. Participants were requested to complete the questionnaire at the hospital just before the medical visit. Details of the questionnaire are shown in the Supplementary material. It was divided into four sections. The first section included demographic data (age, gender), health status perceived by the patient (using seven categories, from “very bad” to “very good”) [17, 18] and education level (categorized as primary, secondary and university studies).

The second section included an initial question focused on basic knowledge on abdominal wall defects using the question Do you come to the medical visit for the assessment of a hernia? with three possible answers (yes, no, I do not know it depends on what the doctor says). Then, the importance assigned to the reasons of medical consultation was evaluated, including “to receive medical advice”, “I already know that I have a hernia, and would like to be operated on as soon as possible”, “to include me in the waiting list for surgery”, “to understand more about my case”, and “to get a prescription of pain medication” (categorized using a 5-point Likert scale from “low importance” to “extremely important”).

The third section explored the patient’s opinion regarding the indication of surgery based on the question Do you think that you should be operated on? with three possible answers (yes, no, I do not know it depends on what the doctor says). In case of an affirmative answer, the next question was Do you think that surgery is the definitive solution? (yes/no). In case of having to undergo hernia repair, the patient had to assess the difficulty of hernia surgery using seven categories from “very easy” to “very difficult”.

The fourth section collected information on the patient’s expectations regarding their future in case of being operated on using 11 statements of the Cardiac Surgery Patient Expectations Questionnaire (C-SPEQ) [19] adapted to hernia surgery. Each statement was scored using a 5-point Likert scale from 1 = “strongly agree” to 5 = “strongly disagree”.

### Data collection

For each patient data collected included results of the study questionnaire and complexity of the abdominal wall defect using criteria proposed by Slater et al. [20], which includes simple and complex midline incisional and ventral hernia, complex abdominal wall defect other than midline hernias, simple inguinal hernia, inguino-scrotal hernia, bilateral inguinal hernia, and no hernia in patients with negative findings on physical examination.

### Statistical analysis

Categorical variables are expressed as frequencies and percentages, and continuous variables as mean and standard deviation (SD). Different comparisons were performed: first, basic knowledge on abdominal wall defects and education level were compared with aspects related to the importance assigned to reasons of medical consultation. Second, responses on whether the patient should be operated on or not, complexity of hernia and education level were compared with the difficulty that the patient attributed to hernia surgery. Third, the mean score of the patient’s expectations was compared with age, gender and education level. The chi-square test or the Fisher’s exact test were used for the comparison of categorical variables, and the Kruskal–Wallis test or the Mann–Whitney *U* test for the comparison of quantitative variables according to conditions of application. Statistical significance was set at *P* < 0.05. The Statistical Package for the Social Sciences (SPSS) version 23.0 (IBM Statistics, Chicago, IL, USA) was used for the analysis of data.

## Results

During the study period, 579 patients were visited for the first time with a presumed diagnosis of an abdominal wall defect, but 130 (22.4%) refused to participate in the study. The remaining 449 patients (27.2% women, mean age 61.5 [16.1] years) agreed to participate and gave written informed consent. Salient data of the patients are shown in Table 1. More than half of the patients (53.9%) had secondary or university studies. On physical examination, inguino-scrotal hernias were found in 11 (2.4%) patients and complex hernias in 57 (12.7%). Twenty (4.5%) patients did not have hernia on physical examination at the time of medical consultation. The patient’s perceived health status was “neither bad nor good” or “good” in 56.6% of cases. Also, more patients considered that hernia repair would be an easy procedure (35.1%) rather than a difficult one (9.8%).

**Table 1:** Clinical characteristics and patients’ opinions regarding hernia surgery

Variables	All patients (*n* = 449)
Men/women (%)	327 (72.8)/122 (27.2)
Age, years, mean (SD)	61.5 (16.2)
Patient’s perceived health status	
Very bad	13 (2.9)
Quite bad	16 (3.6)
Bad	71 (15.8)
Neither bad nor good	128 (28.5)
Good	126 (28.1)
Quite good	67 (14.9)
Very good	15 (3.3)
Missing	13 (2.9)
Education level	
Primary	164 (36.5)
Secondary	156 (34.7)
University	86 (19.2)
Missing	43 (9.6)
Complexity of hernia	
Simple midline incisional hernia	77 (17.1)
Complex midline incisional hernia	31 (6.9)
Simple midline ventral hernia	54 (12.0)
Complex midline ventral hernia	9 (2.0)
Complex abdominal wall defect	17 (3.8)
Simple inguinal hernia	205 (45.7)
Inguino-scrotal hernia	11 (2.4)
Bilateral inguinal hernia	11 (2.4)
No hernia	20 (4.5)
Missing	14 (3.1)
Patient’s perceived difficulty of hernia repair	
The easiest	49 (10.9)
Very easy	28 (6.2)
Quite easy	81 (18.0)
Neither easy nor difficult	181 (40.3)
Quite difficult	29 (6.5)
Very difficult	14 (3.1)
The most difficult	1 (0.2)
Missing	66 (14.7)

**Fig. 1:**
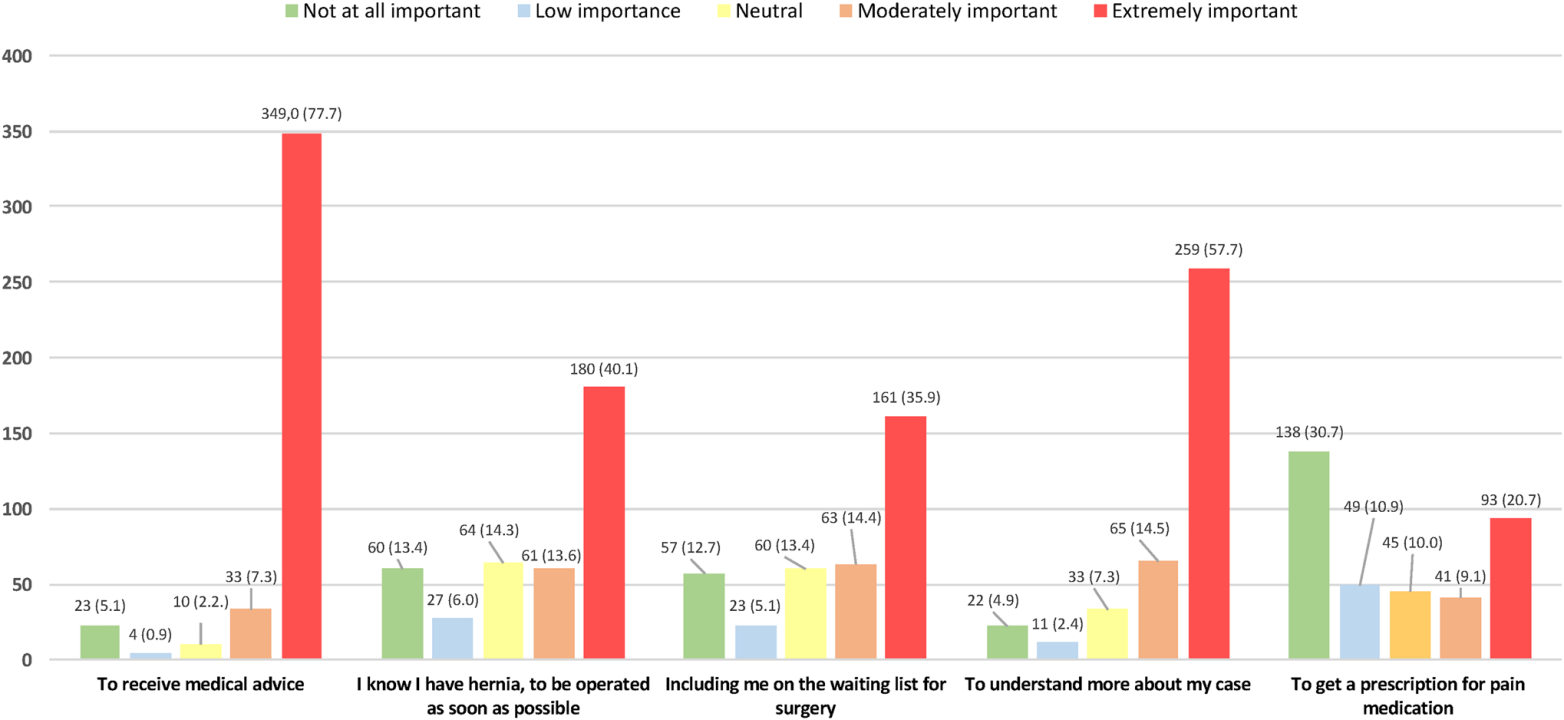
Importance assigned to the reason of medical visit. Absolute frequencies and percentage (%)

Although patients were referred to the abdominal wall surgery unit by their family physicians, 32 (7.1%) patients answered negatively to the question of coming to the visit to assess the presence of a hernia (Table 2). The most important reason of the medical visit was to receive medical advice by 349 (77.7%) of the patients, to be operated on as soon as possible by 180 (40.1%) or to be included in the surgical waiting list by 161 (35.9%). However, more than half of the patients (57.7%) expressed their desire to know more about the disease (Fig. 1). On the other hand, 46.1% of the patients considered that they should undergo a hernia repair and 56.8% that surgery will be a definitive solution for their medical problem (Table 2).

**Table 2:** Results of the patient’s values regarding different aspects of hernia surgery

Variables	Answers to the question			Missing answers
	Yes	No	Depends on the doctor’s opinion	
Do you come to the visit to assess the presence of a hernia?	412 (91.8)	32 (7.1)	5 (1.1)	0
Do you think that you should be operated on	232 (51.7)	9 (2.0)	207 (46.1)	1
Do you think that surgery is the definitive solution?	255 (56.8)	21 (4.7)	NA	173

*NA* not applicable

**Fig. 2:**
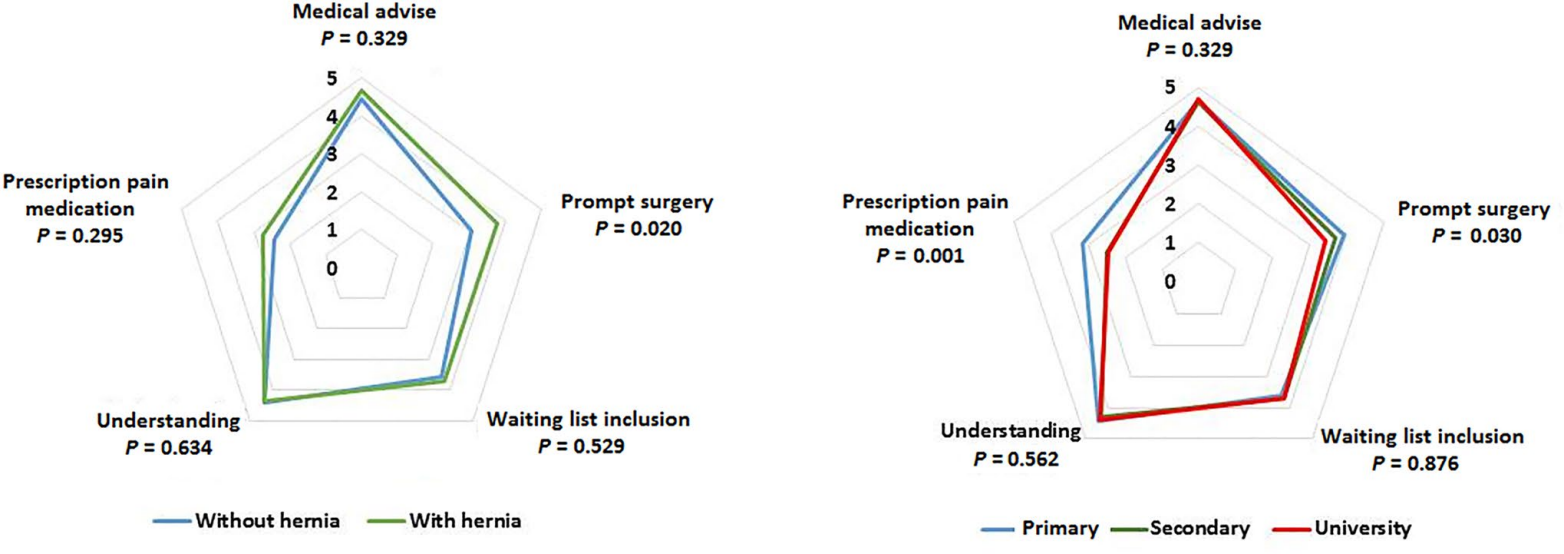
Importance of the different aspects related to medical consultation in relation to the presence/absence of hernia (left panel) and education level (right panel)

As shown in Fig. 2, if the patient knew that he/she had a hernia, to be operated as soon as possible was considered more importantly, with statistically significant differences (*P* = 0.020) as compared with to receive medical advice, inclusion in the waiting list, understanding more about the disease, and get a prescription for pain medication. To be operated on as soon as possible (*P* = 0.030) and to get a prescription for pain medication (*P* = 0.001) were significantly associated with the lower level of education (primary studies).

**Fig. 3:**
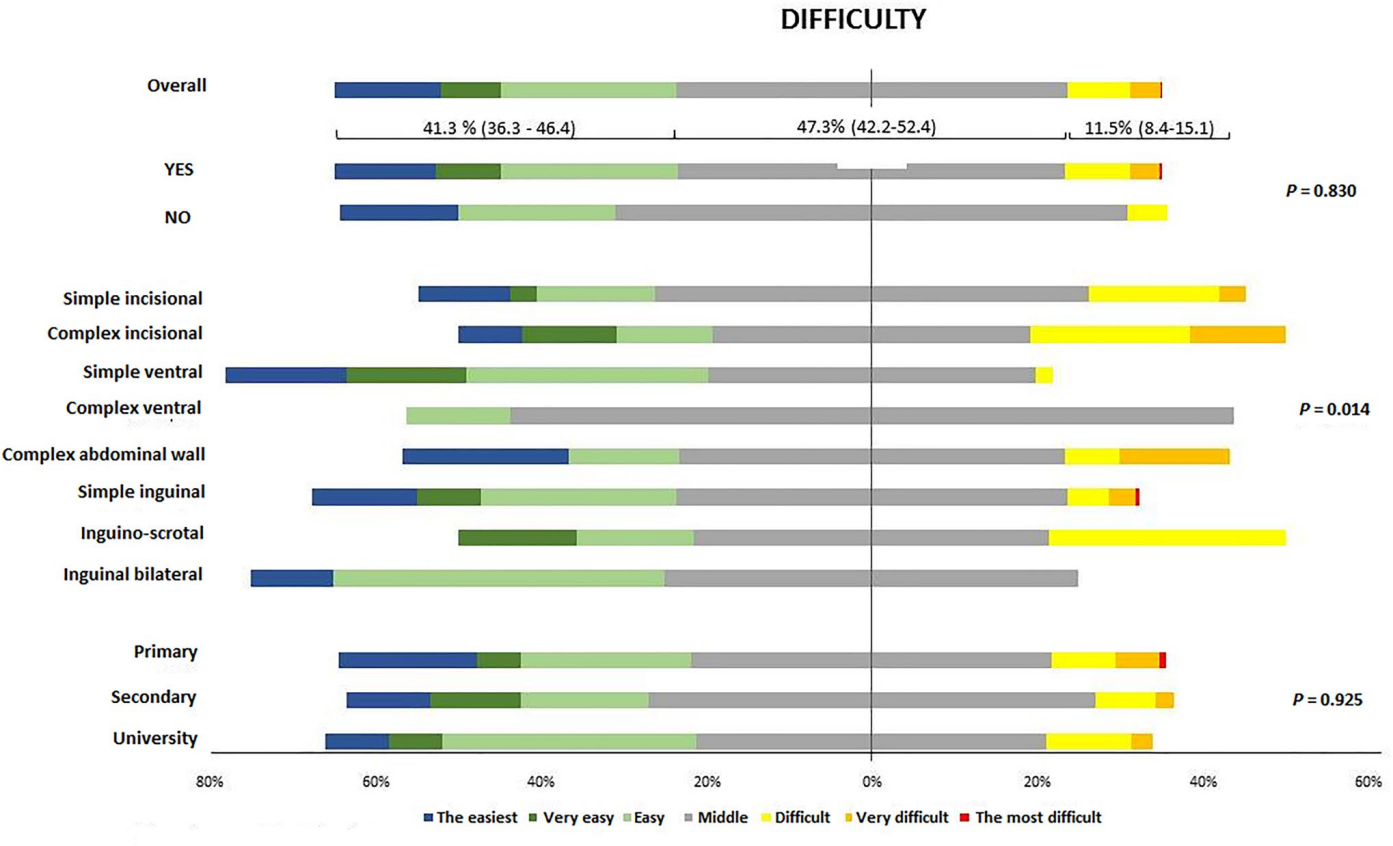
Patient’s assessment of the difficulty of the surgical procedure according to whether or not surgery was considered necessary, complexity of hernia and education level (percentages calculated excluding missing answers)

The comparison of responses regarding whether the patient should be operated on or not, complexity of hernia and education level with the difficulty that the patient attributed to hernia surgery showed that a low percentage of patients considered hernia repair as a difficult surgical procedure (11.5%, 95% confidence interval 8.4–15.1) (Fig. 3 and Table 3). Operations of complex hernias were significantly considered easier procedures (*P* = 0.014) and the education level did not influence upon difficulty of hernia repair (Fig. 3).

**Table 3:** Patient’s perceived difficulty of hernia repair according to different factors

Factors	Patient’s perceived difficulty, number (%)						
	The easiest	Very easy	Quite easy	Middle (neither easy nor difficult)	Quite difficult	Very difficult	The most difficult
Overall (*n* = 383)	49 (12.8)	28 (7.3)	81 (21.1)	181 (47.3)	29 (7.6)	14 (3.6)	1 (0.3)
Surgery							
Yes (*n* = 359)	44 (12.3)	28 (7.8)	77 (21.4)	168 (46.8)	28 (7.8)	13 (3.6)	1 (0.3)
No (*n* = 21)	3 (14.3)	0	4 (19.0)	13 (61.9)	1 (4.8)	0	0
Complexity of hernia							
Simple midline incisional hernia (*n* = 63)	7 (11.1)	2 (3.2)	9 (14.3)	33 (52.4)	10 (15.9)	2 (3.2)	0
Complex midline incisional hernia (*n* = 26)	2 (7.7)	3 (11.5)	3 (11.5)	10 (38.5)	5 (19.2)	3 (11.5)	0
Simple middle ventral hernia (*n* = 48)	7 (14.6)	7 (14.6)	14 (29.2)	19 (39.6)	1 (2.1)	0	0
Complex middle ventral hernia (*n* = 8)	0	0	1 (12.5)	7 (87.5)	0	0	0
Complex abdominal wall defect (*n* = 15)	3 (20)	0	2 (13.3)	7 (46.7)	1 (6.7)	2 (13.3)	0
Simple inguinal hernia (*n* = 182)	23 (12.6)	14 (7.7)	43 (23.6)	86 (47.2)	9 (4.9)	6 (3.3)	1 (0.5)
Inguino-scrotal hernia (*n* = 7)	0	1 (14.3)	1 (14.3)	3 (42.8)	2 (28.6)	0	0
Bilateral hernia (*n* = 10)	1 (10)	0	4 (40)	5 (50)	0	0	0
Education level							
Primary (*n* = 131)	22 (16.8)	7 (5.3)	27 (20.6)	57 (43.5)	10 (7.6)	7 (5.3)	1 (0.8)
Secondary (*n* = 137)	14 (10.2)	15 (10.9)	21 (15.3)	74 (54.0)	10 (7.3)	3 (2.2)	0
University (*n* = 78)	6 (7.7)	5 (6.4)	24 (30.8)	33 (42.3)	8 (10.2)	2 (2.6)	0

**Fig. 4:**
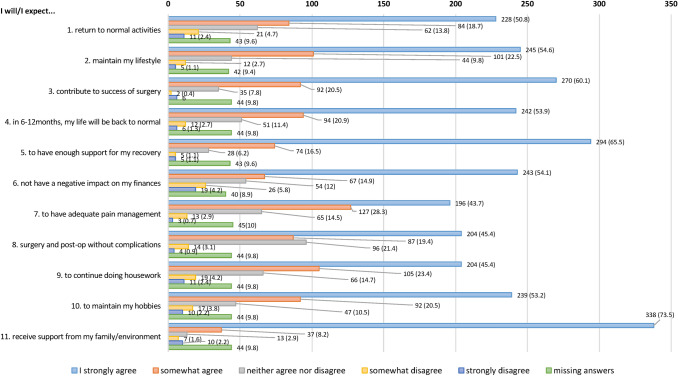
Patient’s expectations in the event of undergoing hernia repair. Absolute frequencies and percentage (%)

Patient’s expectations in the event of undergoing hernia repair are shown in Fig. 4. In general, they were optimistic regarding the results of surgery with return to normal activities and to receive adequate support for recovery. Significant differences in expectations according to age (*P* = 0.273), gender (*P* = 0.092) and education level (*P* = 0.53) were not found.

## Discussion

This study explored the values, preferences and knowledge of patients regarding their pathological condition when referred to the specialist for the first time to assess the indication of hernia repair. The study population was composed mainly by men, with a mean age of 61.5 years, and a self-perceived health status qualified as good or very good. More than half of the patients had completed secondary and/or university studies, and the group of inguino-scrotal hernias and complex abdominal wall hernias accounted for more than 15% of the defects.

Interestingly, although patients were referred by their family physician with a presumed diagnosis of hernia, 7.1% of participants stated that they did not come to the visit because of a hernia, and moreover, the physical examination was unrevealing in 4.1% of patients. Limited knowledge on how to detect herniation on clinical examination, difficulties in palpating the affected area or lack of time at the primary healthcare level may be the reason of these inconsistencies. However, the doctor referring the patients with hernia to the surgeon plays a crucial role in providing the initial information that would help the patients in the SDM process.

In relation to the patient’s values before the clinical visit, to receive medical advice was very important to most patients (77.7%), which agrees with what patients really want or demand from healthcare [11]. However, 40.1% of participants selected to undergo surgery without delay and 35.9% to be included in the surgical waiting list, some preferences that deviate from the shared decision process by suggesting preconception ideas without a clear perspective of the surgeon’s role as responsible for treatment. The high percentage of patients with clear surgical preferences before the medical appointment raises several questions, such as the potential influence of different factors, including previous comments of healthcare professionals who will not be the effectors of treatment, a view of the surgeon’s technical skills for performing an aspect of treatment that cannot be done by other clinicians, information found on internet, or experiences of previous encounters with surgeons in which information provided was mostly limited to surgical technical details. It should be noted that the patient’s preference for being operated without delay was a significantly more common reason in patient who already were aware that they had an abdominal wall defect as well as in those with the lower education level.

Also, 51.7% of patients believed that they should be operated on, 56.8% that surgical repair would be a definitive solution and 11.5% that hernia surgery was a difficult procedure. By contrast, there was a generalized opinion that hernia repair was an easy surgical procedure and this point of view was independent of the patient’s education level. However, practicing surgeons with experience in hernia repair recognize the increasing complexity of abdominal wall surgery due to more challenging cases, newer techniques, tailored approach to such surgery and potential complications and morbidity associated with recurrence rates [21–23]. In addition, the patient’s perception of no surgical difficulty may indirectly reflect the lowest priority assigned to the real cost of treatment in the framework of public and universal healthcare systems, such as that of our hospitals, as well as a certainty that operations will run without complications together with a high perception of safety within the current surgical setting [24]. Strategies to improve the patient’s perspectives are necessary for several reasons, first, because a false feeling of safety may negatively affect adherence to prehabilitation programmes before surgery and, second, because an adequate patient’s perspective is an essential element in the assessment of systems and processes for improving the quality of care [25].

In agreement with data reported in other medical disciplines [4, 12, 13], the use of adequate information is crucial to help patients for clarifying preconceptions and myths related to hernia surgery. A low level of understanding regarding preferences, knowledge and expectations based on complexity of hernia may be related to the education level, personal experiences, knowledge and attitude of the referral family physician and many other factors that are needed to be explored in future studies.

The present findings should be interpreted taking into account some limitations of the study, such as difficulties to put the patients’ responses into appropriate context due to the large range of patients included in the cohort from a variety of hernia types to a variety of hernia severity, the relatively small sample size and the limited external validity for the generalizability of results to other healthcare systems and sociocultural settings. However, some of the data here reported may be applicable to patients with abdominal wall defects and may constitute a reference framework for future studies focused on PRK in surgical patients with hernia. New strategies favouring clear and simple messages of abdominal wall defects are necessary to better inform patients and enhance their proactive role in working together with surgeons to make decisions and select the best treatment approach. In our opinion, improving communication skills of physicians referring patients for hernia surgery to provide more comprehensive information of the disease, better control of websites sometimes offering patients ‘unrealistic hernia solutions’, and stronger commitment of managers for high priority programs related to hernia surgery in the framework of the different healthcare systems are some aspects that would require critical/urgent attention to improve PRK.

We conclude that PRK of patients referred for the first time to an abdominal wall surgery unit with a presumed diagnosis of hernia is quite poor and there is still a long way towards improving knowledge of hernia surgery. Optimization of communication between primary and specialized levels of care and with the patients themselves would increase empowerment of patients with hernia undergoing surgical repair, as well as improvement in the shared decision-making process for a highly prevalent surgical condition, such as abdominal wall defects.

## Supplementary Information

Below is the link to the electronic supplementary material.

Supplementary file1 (DOCX 26 KB)
