# Supplementary material for: Understanding patient-reported knowledge of hernia surgery: a quantitative study
Source: Hernia. 2021 Oct 20;26(3):761–8. doi: 10.1007/s10029-021-02521-6 (PMC9200871; doi:10.1007/s10029-021-02521-6)
Supplement: Supplementary file 1 — Supplementary file1 (DOCX 26 KB) [file 10029_2021_2521_MOESM1_ESM.docx]

Patient code:

**RAPID QUESTIONNAIRE OF THE PATIENT’S PREFERENCES AT THE ABDOMINAL WALL SURGICAL UNIT CONSULTATION**

1. Demographic data (age, gender):
2. Health status perceived by the patient: *How* *would you rate your* *health status?*

| Very bad | Quite  bad | Bad | Neither bad nor good | Good | Quite  good | Very good |
| --- | --- | --- | --- | --- | --- | --- |

1. Education level: *What is your level of studies?* Primary/Secondary/University
2. *Do you come to the medical visit to assess the presence of a hernia*?

- Yes
- No
- It depends on what the doctor says

1. Mark the importance of each of the following aspects related to the medical consultation (1: low importance; 5: extremely important)

|  | 1 | 2 | 3 | 4 | 5 |
| --- | --- | --- | --- | --- | --- |
| To receive medical advice |  |  |  |  |  |
| I already know that I have a hernia, but to be operated on as soon as possible |  |  |  |  |  |
| Including me on the waiting list for surgery |  |  |  |  |  |
| To understand more about my case |  |  |  |  |  |
| To get a prescription for pain medication |  |  |  |  |  |

1. *Do you think that you should be operated on?*

- Yes
- No
- I do not know, it depends on what the doctor says
  1. If you have answered YES, *Do you think that surgery is the definitive solution?*
- Yes
- No

| In the event that you should undergo surgery, how do you think that the hernia repair will be? | If you do not have a hernia, how do you think that the hernia repair will be? |
| --- | --- |
| - The easiest - Very easy - Quite easy - Neither easy nor difficult - Quite difficult - Very difficult - The most difficult | - The easiest - Very easy - Quite easy - Neither easy nor difficult - Quite difficult - Very difficult - The most difficult |

In the event that I should undergo surgery… (mark the corresponding box with a cross)

|  | Strongly agree  (1) | Somewhat agree (2) | Neither agree nor disagree (3) | Somewhat disagree (4) | Strongly disagree (5) |
| --- | --- | --- | --- | --- | --- |
| 1. I will easily return to normal activities (work/studies …) |  |  |  |  |  |
| 1. I will able to maintain my lifestyle |  |  |  |  |  |
| 1. I expect to do something to contribute to the success of surgery |  |  |  |  |  |
| 1. I expect that in 6-12 months, my life will be back to normal |  |  |  |  |  |
| 1. I expect to have enough support for   my recovery |  |  |  |  |  |
| 1. It will not have a negative impact on my finances |  |  |  |  |  |
| 1. I expect to have adequate pain management |  |  |  |  |  |
| 1. Surgery and the postoperative period will run without complications |  |  |  |  |  |
| 1. I will be able to continue doing housework |  |  |  |  |  |
| 1. I expect to maintain my hobbies |  |  |  |  |  |
| 1. I expect to receive support from my family/environment |  |  |  |  |  |
